# A rapid and visual detection assay for Senecavirus A based on recombinase-aided amplification and lateral flow dipstick

**DOI:** 10.3389/fcimb.2024.1474676

**Published:** 2024-10-23

**Authors:** Yiwan Song, Yiqi Fang, Shuaiqi Zhu, Weijun Wang, Lianxiang Wang, Wenxian Chen, Yintao He, Lin Yi, Hongxing Ding, Mingqiu Zhao, Shuangqi Fan, Zhaoyao Li, Jinding Chen

**Affiliations:** ^1^ College of Veterinary Medicine, South China Agricultural University, Guangzhou, China; ^2^ Key Laboratory of Zoonotic Disease Prevention and Control of Guangdong, South China Agricultural University, Guangzhou, China; ^3^ Wen’s Group Academy, Wen’s Foodstuffs Group Co., Ltd., Guangzhou, Guangdong, China

**Keywords:** Senecavirus A, recombinase-aided amplification, lateral flow dipstick, sensitivity, specificity, visual detection

## Abstract

**Background:**

Senecavirus A (SVA) is a newly pathogenic virus correlated with the acute death of piglets and vesicular lesions in pigs. The further prevalence of SVA will cause considerable economic damage to the global pig farming industry. Therefore, rapid and accurate diagnostic tools for SVA are crucial for preventing and controlling the disease.

**Methods:**

We designed multiple primer pairs targeting the most conserved region of the SVA *3D* gene and selected those with the highest specificity. Nfo-probes were subsequently developed based on the highest specificity primer pairs. Subsequently, the recombinase-assisted amplification (RAA) reaction was completed, and the reaction temperature and duration were optimized. The RAA amplicons were detected using a lateral flow device (LFD). Finally, a rapid and intuitive RAA-LFD assay was established against SVA.

**Results:**

The SVA RAA-LFD assay can be performed under reaction conditions of 35°C within 17 minutes, with results observable to the naked eye. We then evaluated the performance of this method. It exhibited high specificity and no cross-reaction with the other common swine pathogens. The lowest detectable limits of this method for the plasmid of pMD18-SVA-3D, DNA amplification product, and viral were 3.86×10^1^ copies/µL, 8.76×10^-7^ ng/µL, and 1×10^0.25^ TCID_50_/mL, respectively. A total of 44 clinical samples were then tested using the RAA-LFD, PCR, and RT-qPCR methods. The results demonstrated a consistent detection rate between the RAA-LFD and RT-qPCR assays.

**Conclusion:**

The SVA RAA-LFD assay developed in our study exhibits excellent specificity, sensitivity, and time-saving attributes, making it ideally suited for utilization in lack-instrumented laboratory and field settings.

## 1 Introduction

Senecavirus A (SVA) is a single-stranded, positive RNA virus belonging to the genus *Senecavirus* within the family *Picornaviridae* (Adams et al., 2015; King et al., 2012). SVA is among the pathogens associated with vesicular diseases in pigs, characterized primarily by acute mortality in neonatal piglets and the development of vesicular ulcers in the nostrils, hoof coronets, and coronary arteries of adult pigs (Joshi et al., 2016; Vannucci et al., 2015). Consequently, distinguishing disease infected by SVA from other swine vesicular diseases such as foot-and-mouth disease (FMD), swine vesicular disease (SVD), and vesicular stomatitis (VS) based solely on clinical symptoms has been a challenging task. Furthermore, this disease lacks distinct seasonal epidemic trends, posing additional challenges for prevention and control efforts (Hales et al., 2008). The SVA genome is approximately 7300 bp, comprising a 5’ untranslated region (5′ UTR), an open reading frame (ORF), and a 3’ untranslated region (3′ UTR) (Lin et al., 2009). The ORF consisted of a leader protein (L) and three precursor proteins (P1, P2, and P3) (Adams et al., 2016; Hales et al., 2008; Venkataraman et al., 2008). Subsequently, these regions are further processed by the virus-encoded cysteine protease (3C^pro^) to form mature functional viral proteins (L-VP4-VP2-VP3-VP1-2A-2B-2C-3A-3B-3C-3D) (Hales et al., 2008; Leme et al., 2015).

In 2002, SVA was identified in cultivating transformed embryonic retinal cell lines (C6 cells) grown at a U.S. research company (Knowles et al., 2006). Following isolation and purification, the virus was named SVV 001 (Hales et al., 2008). Since the identification of SVV001, several studies have shown that it is closely associated with subsequent sporadic cases of porcine idiopathic vesicular disease (PIVD) in the USA (Bracht et al., 2016; K. et al., 2012) and Canada (Pasma et al., 2008). Interestingly, a series of outbreaks of PIVD occurred in Brazil between 2014 and 2015, subsequently confirmed to be caused by SVA infection. This confirmed that SVA is the pathogen responsible for swine vesicular disease (Vannucci et al., 2015). Since then, two outbreaks of SVA infections have been reported in the Brazilian region in 2018 and 2020 (Leme et al., 2019; Vieira et al., 2022). Additionally, several countries within the areas of Europe, Southeast Asia, and Asia have published reports of SVA infections (Arzt et al., 2019; Hause et al., 2016; Saeng-chuto et al., 2017; Sun et al., 2017; Wu et al., 2017; Xu et al., 2017). The above situation implies that SVA is gradually prevalent, which will pose a severe economic threat to the swine industry worldwide. Therefore, the prevention and control of SVA is crucial to avoid further dissemination.

Currently, a number of detection methods for SVA have been developed in the laboratory, including PCR (Pinheiro-de-Oliveira et al., 2019; Zhang et al., 2019), qPCR (Mu et al., 2020; Zhang et al., 2019), and ELISA (Dvorak et al., 2017; Yang et al., 2012). These methods are designed to prevent SVA infections from affecting the economy of the pig farming industry. However, these detection methods require a thermocycler for target gene amplification or proficient laboratory techniques, rendering them unsuitable for laboratories with limited infrastructure or field inspections.

Isothermal nucleic acid amplification is a technique developed in recent years that uses specific enzymes to amplify target genes under constant temperature conditions, with methods including methods such as Loop-mediated isothermal amplification (LAMP) (Peng et al., 2024; Wang et al., 2024) and recombinase-aided amplification (RAA) (Feng et al., 2024; Zhou et al., 2024). These methods require only a constant-temperature heating block or even body heat to achieve target gene amplification, significantly reducing the reaction time and complexity of nucleic acid amplification (Kolm et al., 2019). However, the design of the LAMP primer is more complex and technically challenging. In contrast, the primer design for RAA is straightforward, requiring only the control of the target gene length within 500 bp and the design of two primers with lengths of 30~35 bp. The RAA reaction primarily utilizes recombinase, single-stranded binding proteins (SSBs), and DNA polymerase with strand displacement activity to initiate the reaction. It can complete nucleic acid amplification in less than 40 minutes. This method has been used to detect a variety of pathogens (Hai, 2010; He et al., 2021; Li et al., 2023; Wu et al., 2022; Zhao et al., 2024b).

Lateral flow dipstick (LFD) is a technique for rapidly detecting nucleic acid samples based on capillary chromatography, molecular hybridization, and colloidal gold (Jaroenram et al., 2009). The method is utilized to detect biomarkers in nucleic acid samples, requiring neither specialized equipment nor operational expertise, thus rendering it accessible to a broad range of users (Jaroenram and Owens, 2014). The combination of RAA and LFD is based on the principle that the RAA reaction forms amplified products labeled with biotin and specific fluorescent antibodies, which then interact with the colloidal gold-labeled antibodies in the LFD, further cross-linking with biotin on the test (T)-line, ultimately achieving the visual detection of the target product (Chow et al., 2008). The RAA-LFD assay has been successfully used for the detection of a variety of pathogens (Bienes et al., 2022; Homklinkaew et al., 2023; Hou et al., 2022; Li et al., 2022).

In this study, the most optimal RAA primers and probes were designed for the most conserved region of the SVA *3D* gene. The RAA products were detected using LFD, and a highly sensitive and specific SVA RAA-LFD rapid visualization assay was established. This method overcomes the difficulties of ordinary PCR and qPCR, which require expensive nucleic acid amplifiers. It provides a reliable method for the initial screening and detection of SVA in ordinary laboratories and the field.

## 2 Materials and methods

### 2.1 Viruses

SVA, foot and mouth disease virus (FMDV), african swine fever (ASFV), porcine circovirus type 2 (PVC2), japanese encephalitis virus (JEV), classical swine fever virus (CSFV), and porcine parvovirus (PPV) were obtained from the Laboratory of Veterinary Microbiology and Immunology, South China Agricultural University.

### 2.2 Clinical swine samples

In 2018, forty-four clinical swine samples suspected of SVA infection were obtained from multiple pig farms in Guangzhou and were stored at -80°C for further utilization. RNA was extracted from each clinical sample using the E.Z.N.A.^®^ FFPE RNA Kit (Omega Bio-Tek, Inc., Connecticut, USA) and stored at -80°C in our laboratory.

### 2.3 Design and screening of primers

According to previous studies conducted in our laboratory and the research conducted by Li et al (Li et al., 2019), the most conserved region of SVA is located within the *3D* gene, which can be a suitable target for RAA amplification. Thirty-seven SVA sequences were obtained from the National Center for Biotechnology Information (NCBI) (GenBank accession numbers: MF615501.1, MF615506.1, MF615507.1, MF615508.1, MF615509.1, MF189001.1, MF189000.1, KR063107.1, MF460448.1, MH885100.1, MH885099.1, MH634510.1, MH634508.1, MH634506.1, MH634518.1, MH634522.1, MH634516.1, MH716015.1, MHS88717.1, MH779611.1, MH817445.1, MH316117.1, MH316116.1, MK357117.1, MN781984.1, MN781983.1, MN781981.1, KRO63107.1, ON369394.1, ON868377.1, KR063109.1, MZ818785.1, MZ375462.1, KY486166.1, KY486165.1, MZ395819.1, MZ733980.1). The *3D* gene regions of the above sequences were aligned using MegAlign 7.1 (DNAStar, USA) to confirm the most conserved region. Subsequently, six primer pairs were designed within this region using Oligo 7.0 (Molecular Biology Insights, Inc, USA) and Primer Premier 5 (Premier Biosoft International, Canada) (**Table 1**). The specificity of the six pairs of primers for SVA was verified by the RAA reaction provided by the RAA nucleic acid amplification kit based on agarose gel electrophoresis (AGE) (Jiangsu Qitian Gene Biotechnology Co., Ltd., Jiangsu, China), and the most specific primers were screened. Components including 25 μL rehydration buffer V, 2.0 μL forward primer (10 μM), 2.0 μL reverse primer (10 μM), and 17.5 μL nuclease-free water were sequentially added to the lyophilized enzyme base reaction unit in the kit. Then, the walls of the tubes were flicked by hand and centrifuged transiently for 10 seconds. To stimulate the reaction, 2.5 μL magnesium acetate was introduced into the inside of each tube cap, and 1μL of genomic DNA was added as a template. Additionally, the ddH2O and positive samples were added to the separately formulated reaction system as negative and positive controls, respectively. After 40 minutes incubation period at 37°C, 50 μL of chloroform and phenol mixture (1:1) was added to purify the amplicons. Finally, the supernatant was extracted for analysis using 2% AGE.

**Table 1:** The primers of the 3D-based RAA basic reaction for SVA were used in this study.

Primer	Sequences (5’-3’)	Length (bp)
SVA RAA-F1	TCTGGTTGGTACGGATTACGATCTGGACTTCA	100
SVA RAA-R1	GAAGACAGAACCCTTGTTGGCAGGAGTCATCT	
SVA RAA-F2	TCTAAACACACTGCCAACGTCCCTTATCAACC	284
SVA RAA-R2	TAGTCACCGTCTAAGAATTTTTGGATTTGCAT	
SVA RAA-F3	TAAACACACTGCCAACGTCCCTTATCAACCTC	282
SVA RAA-R2	TAGTCACCGTCTAAGAATTTTTGGATTTGCAT	
RAA SVA-F4	AAACTGGGGTACAAGATGACTCCTGCCAAC	212
SVA RAA-R4	GCCAACATAGAAACAGATTGCAGCTTCTCG	
SVA RAA-F5	TCTGGTTGGTACGGATTACGATCTGGACTT	99
SVA RAA-R5	AAGACAGAACCCTTGTTGGCAGGAGTCATC	
SVA RAA-F6	GCCAAACTGGGGTACAAGATGACTCCTGCCAACA	216
SVA RAA-R6	AGCCAACATAGAAACAGATTGCAGCTTCTCGAGT	

### 2.4 Establishment of the SVA RAA-LFD assay

**Figure 1:**
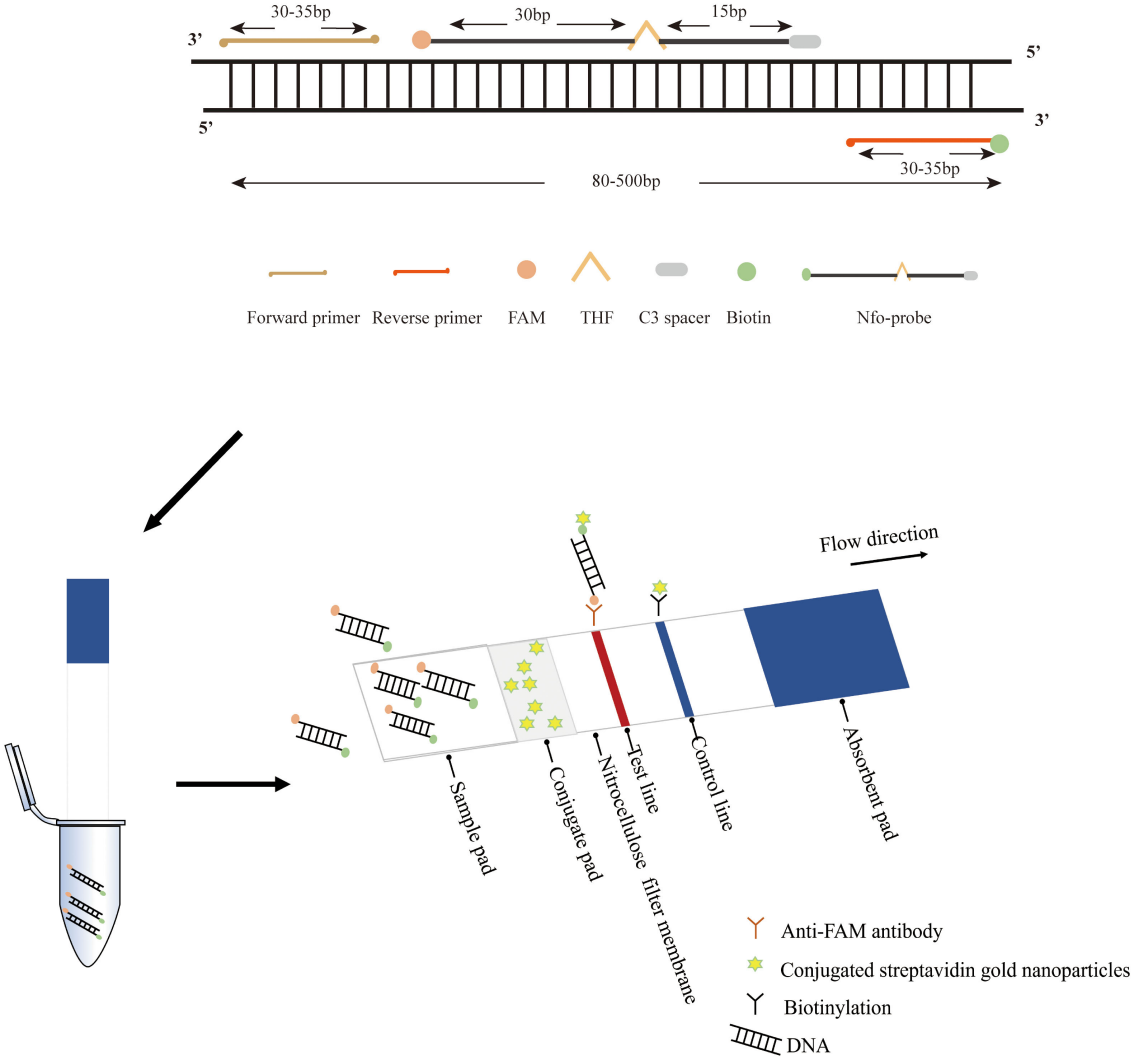
The principle of RAA primers, nfo-probe, and the RAA-LFD assay.

Following the primer screening, a specific probe was developed and situated within the middle of the primer pairs. This probe was labeled with 6-carboxy-fluorescein (FAM) at the 5’ end, modified with a polymerase extension blocking group (C3 spacer) at the 3’ end, and integrated with a tetrahydrofuran abasic-site mimic (THF) moiety positioned 30 nucleotides away from the 5’ end. Additionally, the reverse primer’s 5’ end was biotin-labeled. All primers were synthesized by Sangon Biotech (Sangon Biotech (Shanghai) Co., Ltd, Shanghai, China). The RAA basic reaction for LFD was performed according to instructions provided in the nfo-based RAA nucleic acid amplification kit (Jiangsu Qitian Gene Biotechnology Co., Ltd., Jiangsu, China). The nfo-based RAA reaction system was based on the AGE-based RAA reaction volume with the addition of 0.6 μL of fluorescent probe (10 μM) and an adjusted volume of nuclease-free water (16.9 μL). The reaction was carried out under the same time and temperature conditions, following 10 μL of nfo-based RAA amplicons were taken and diluted with 50 μL of PBS and later inserted into the LFD strips (Nanjing Wobo Biotechnology Co., Ltd., Nanjing, China) for 2 minutes. The design of RAA primers, Nfo-probe, and the principle of the RAA-LFD assay are illustrated in **Figure 1**.

### 2.5 Optimization of the SVA RAA-LFD assay

Initially, the RAA basic reaction was conducted with a fixed reaction time of 40 minutes at a gradient temperature from 34°C to 40°C. The results were visualized using a nucleic acid test strip. The test strips were positioned against a white backdrop, and the T-lines were digitally outlined using ImageJ 2 software (National Institutes of Health; Bethesda, MD, USA) to quantify color intensity. This process was conducted three times, and the collected data were subsequently inputted into GraphPad 8.0 software (Graph Pad Software, La Jolla, CA, USA) to generate a line graph illustrating the grayscale values of the T-lines at the different reaction temperatures. Subsequently, the RAA basic reaction was executed at the optimal temperature determined in the preliminary step, with reaction times varying from 5 to 40 minutes. The results were visualized using a nucleic acid test strip. Applying the methodology above, a line graph was formulated to assess the grayscale values of the T-line at various reaction durations.

### 2.6 Construction of the recombinant plasmid

The SVA *3D* gene was amplified by PCR with designed primers (SVA 3D-F: 5’-TGATGACTGAGCTAGAGCCTGG-3’, SVA 3D-R: 5’-TCGAACAAGGCCCTCCATCTTG-3’). The amplicon was ligated to pMD18-T Vector (Takara Biomedical Technology Co., Ltd., Japan) and transformed into *Escherichia coli* DH5α. The pMD18-SVA-3D plasmid was subsequently confirmed in LB solid medium containing 1‰ ampicillin and expanded in LB broth for overnight incubation at 37°C. Finally, the plasmid was extracted using the Plasmid Mini Kit II (Omega Bio-Tek, Inc., Connecticut, USA) according to the manufacturer’s instructions. A NanoDrop™ 1000 Spectrophotometer (Thermo Fisher Scientific, WymanStreet, USA) was used to measure the concentration of the extracted plasmid and calculate copies using the following equation: Copy number per unit (copies/µL) = (concentration of DNA (ng) × 6.022 × 10^23^)/(entire length of template × 10^9^ × 650) (dsDNA copy number calculator (uri.edu).

### 2.7 Analysis of the specificity and sensitivity of the SVA RAA-LFD assay

SVA and other laboratory-preserved viruses were used as templates to validate the specificity of the developed assay. The lowest detection limit of SVA RAA-LFD was determined using ten-fold serial dilutions of pMD18-SVA-3D plasmid, DNA amplification products, and viral fluid at a concentration of 10^8.25^ TCID_50_/mL as templates. PCR and RT-qPCR methods were employed as parallel experiments for comparison.

### 2.8 RCR assay

The reaction system for PCR (Nanjing Vazyme Biotech Co., Ltd., China) is as follows: 25 µL of 2×Phanta Max Buffer, 1µL of dNTP Mix (10 mM each), 1µL of Phanta Max Super-Fidelity DNA Polymerase, 2 µL of forward primer (10 µM), 2 µL of reverse primer(10 µM), 1 µL of DNA template, and 19 µL of ddH2O. SVA specific forward primer: SVA PCR-F (5’-CAAGGAATTTGAATATGACATGG-3’) and reverse primer: SVA PCR-R (5’-GCAGCTTCTCGAGTAGTGTTCC-3’) were described previously (Qian et al., 2016). Amplicon product size was 298 bp. The thermal cycling conditions: one cycle at 95°C for 2 min; 35 cycles at 95°C for 15 s, 57°C for 30 s, and 72°C for 30 s, and one cycle at 72°C for 5 min. Amplicon products were analyzed by 2% AGE.

### 2.9 RT-qPCR assay

The reagent HiScript II Q Select RT SuperMix for qPCR (Nanjing Vazyme Biotech Co., Ltd., China) was used to reverse transcribe RNA into cDNA. 9 µL 2 × ChamQ SYBR qPCR Master Mix. The reaction system for qPCR is as follows: 5 µL 2 × ChamQ SYBR qPCR Master Mix (Nanjing Vazyme Biotech Co., Ltd., China), 0.2 µL forward primer (10 µM), 0.2 µL reverse primer (10 µM), 1 µL of DNA template, and 3.6 µL of ddH2O. SVA specific forward primer(SVA qPCR-F: 5’-GGGTAACACTGACACCGATTT-3’) and reverse primer (SVA qPCR-R: 5’-TCGAGATCGATCAAACAGGAAC-3’) were designed based on the *VP1* gene region with a length of 87 bp (Bracht et al., 2016). The thermal cycling conditions: 48°C for 5 min, 95°C for 10 min, 40 cycles of 95°C for 5 sec, and 60°C for 1 min.

### 2.10 Analysis of repeatability of the SVA RAA-LFD assay

Several concentrations of the pMD18-SVA-3D plasmid were used as templates to assess the reproducibility of the established assay, including 3.86 × 10^4^ copies/µL (strongly positive), 3.86 × 10^2^ copies/µL (moderately positive) and 3.86 × 10^1^ copies/µL (weak positive). The experiment was repeated three times.

## 3 Results

### 3.1 Design and screening of SVA RAA primers

**Figure 2:**
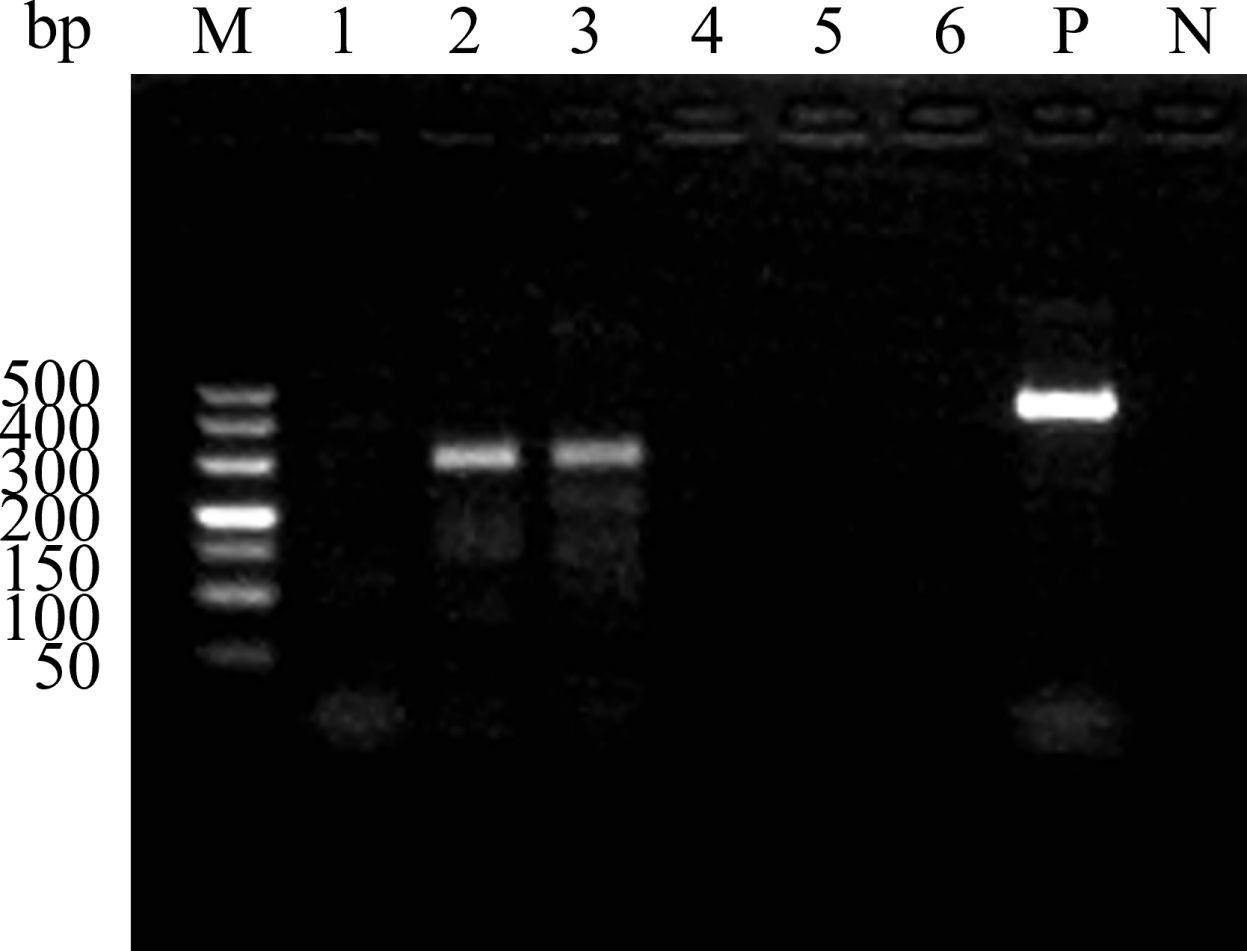
Amplification results of the RAA primer pairs. Serial number 1 to 6 are SVA RAA-F1/R1, SVA RAA-F2/R2, SVA RAA-F3/R2, SVA RAA-F4/R4, SVA RAA-F5/R5, and SVA RAA-F6/R6. M, 500 bp DNA marker; P, Positive control; N, Negative control.

The six candidate primers were evaluated using the RAA basic assay, conducted at 37°C for 40 minutes, with the GD-SVA2-2018 strain as the template. The 2%AGE results (**Figure 2**) demonstrated that the primer pair SVA RAA-F2/R2 produced the brightest specific bands compared to the other groups. These findings indicate that the SVA RAA-F2/R2 primer pair exhibits the highest specificity. Consequently, the SVA RAA-F2/R2 primer pair was selected for further research in the RAA-LFD assay.

### 3.2 Establishment of initial SVA RAA-LFD assay

**Figure 3:**
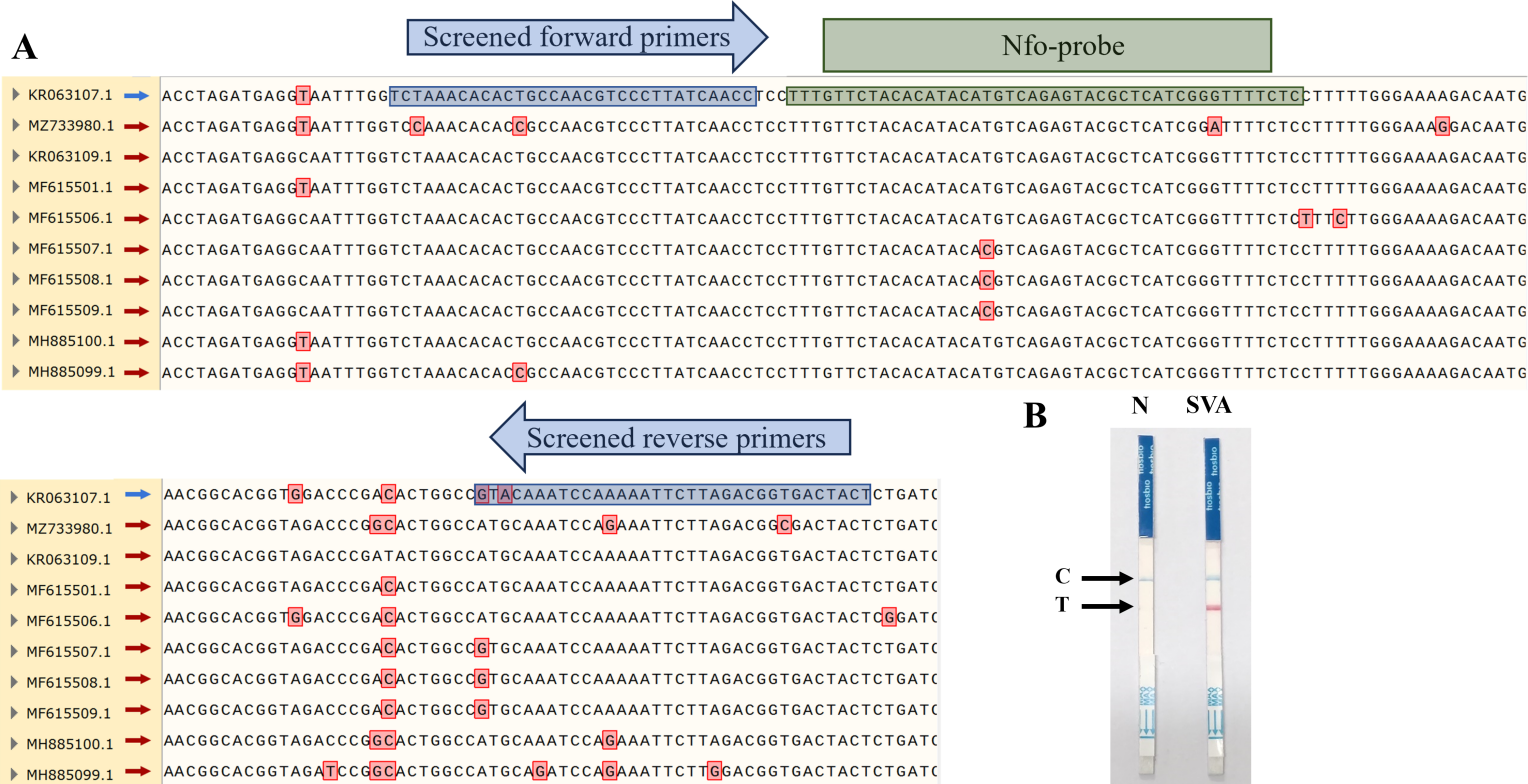
Partial results of multiple sequence alignment for SVA *3D* gene and establishment of SVA RAA-LFD assay. **(A)** Red boxes represent nucleotide mutation sites in the alignment results. Blue boxes represent the binding sites of screened primers. Green boxes represent the binding sites of the nfo-probe. **(B)** Preliminary results of RAA-LFD for SVA detection.

To establish the SVA RAA-LFD assay, we have successfully designed an ideal fluorescent probe according to the gene sequence amplified by the SVA F2/R2 primer pair, nfo-probe (5’-6-FAM-TTTGTTCTACACATACATGTCAGAGTACGC-THF-CATCGGGTTTTCTCC-C3 spacer-3’) (**Figure 3A**). This design fully complies with the criteria for a good nfo-probe. The 5’ end of the reserve primer SAV RAA-R1 was also modified with biotin as RAA-FB: 5’-(Biotin) TAGTCACCGTCTAAGAATTTTTGGATTTGCAT-3’. Subsequently, the nfo-probe and modified and screened primers were used in the nfo-based RAA basic reaction, and LFD detected the amplicons. The results (**Figure 3B**) showed that the control (C)-line in both the negative control and the positive test groups was blue, indicating that the test strip was valid. The T-line in the negative control group showed no color, and the T-line in the SVA experimental group was red, indicating that the test result was positive and that the SVA RAA-LFD assay was initially established.

### 3.3 Optimization of SVA RAA-LFD reaction conditions

**Figure 4:**
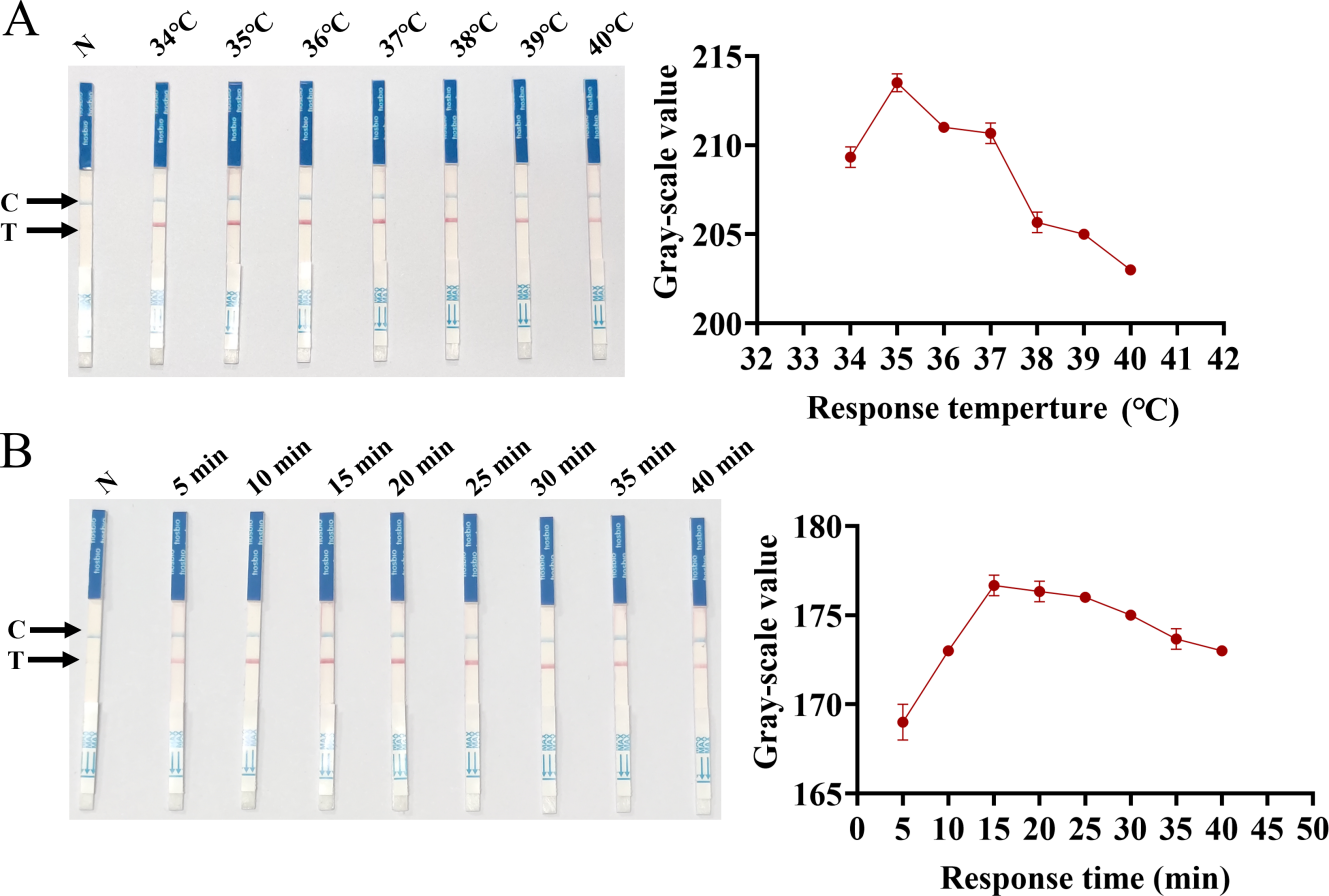
Optimization of SVA RAA-LFD reaction conditions. **(A)** Optimization of temperature for the RAA basic reaction. **(B)** Optimization of time for the RAA basic reaction.

To achieve the optimal reactivity performance of the SVA RAA-LFD assay, the preferred temperature and time for the RAA basic reaction were evaluated. RAA basic reactions were carried out at seven different temperatures from 34°C to 40°C (34°C, 35°C, 36°C, 37°C, 38°C, 39°C, and 40°C) for 40 minutes in a thermostatic. Subsequently, the RAA basic reaction target amplicon was detected by LFD under different temperature gradients. The results indicate that the T-lines of all test strips appeared red, demonstrating the effectiveness of the test strips within the temperature gradients. The result of the gray value analysis of the T-line (**Figure 4A**) showed that 35°C was the optimal reaction temperature for the SVA RAA-LFD assay. Furthermore, the highest value gray value analysis of the T-line was observed at 15 min (**Figure 4B**), indicating that 15 minutes was the optimal reaction time for the RAA basic reaction.

### 3.4 Analytical specificity of the SVA RAA-LFD assay

**Figure 5:**
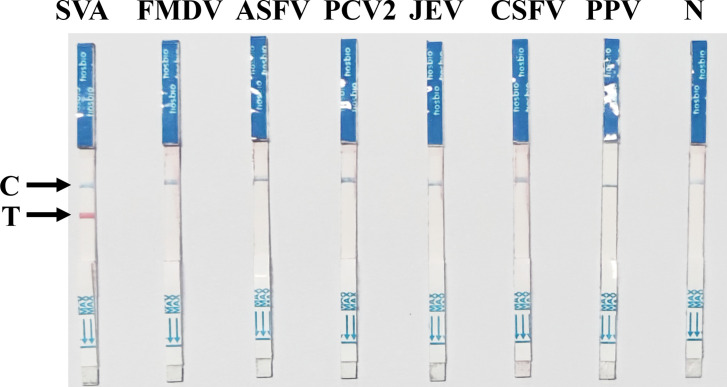
Specificity of the SVA RAA-LFD assay. The nucleic acids of a batch of pathogens, including FMDV, ASFV, PCV2, JEV, CSFV, and PPV, were used to evaluate the specificity of the SVA RAA-LFD assay.

As shown in **Figure 5**, in the analytical specificity analysis, the SVA RAA-LFD only produced visible red bands on the T-line for SVA. None of the other clinically important porcine pathogens, such as FMDV, ASFV, PCV2, JEV, CSFV, and PPV, produced visible bands on the T-line. The specificity of the SVA RAA-LFD assay was further validated by three independent technicians, confirming its high specificity.

### 3.5 Analytical sensitivity of the SVA RAA-LFD assay

**Figure 6:**
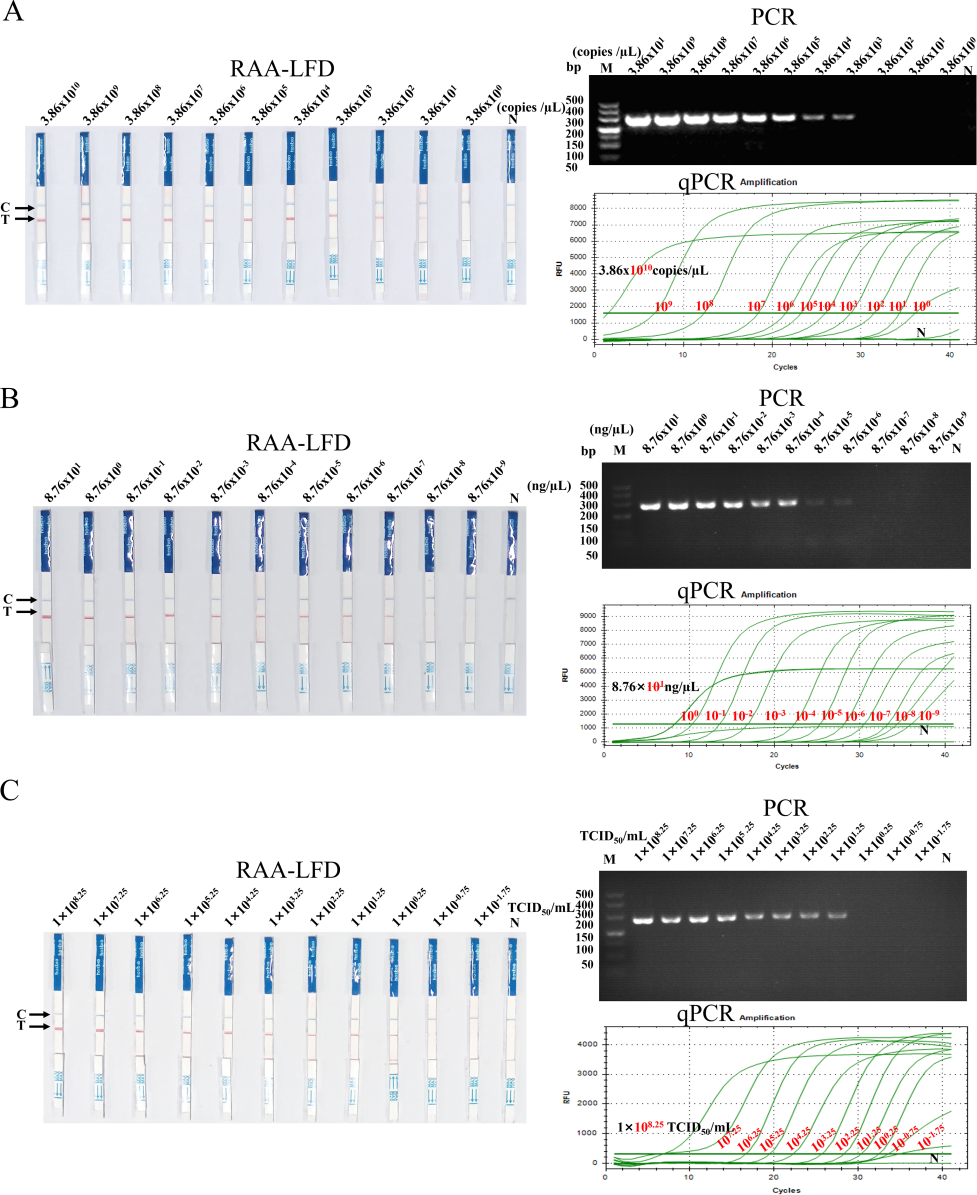
Sensitivity of the SVA RAA-LFD assay. **(A)** The results of detecting serially diluted pMD18-SVA-3D plasmids using the three methods. **(B)** The results of detecting serially diluted SVA DNA amplification product using the three methods. **(C)** The results of detecting serially diluted SVA viral fluid using the three methods. M: 500 bp DNA marker, N: Negative control, Cycles>35: Negative.

To analyze the sensitivity of SVA RAA-LFD detection, this method was used to detect SVA pMD18-SVA-3D plasmids, DNA amplification products, and viral diluted tenfold (3.86×10^10^~3.86×10^0^ copies/µL, 8.76×10^1^~8.76×10^-9^ ng/µL, and 1×10^8.25^~1×10^-1.75^ TCID_50_/mL, respectively). PCR and RT-qPCR were performed as parallel experiments. The results (**Figure 6**) showed that the lowest concentration of template detection of RAA-LFD for SVA plasmids (**Figure 6A**), DNA amplification product (**Figure 6B**), and viral fluid (**Figure 6C**) were 3.86×10^1^ copies/µL, 8.76×10^-7^ ng/µL, and1×10^0.25^ TCID_50_/mL, respectively. The sensitivity of the SVA RAA-LFD assay was significantly higher than that of the PCR assay, yet slightly less than 10 times that of the RT-qPCR assay. However, the sensitivity of this method in detecting the plasmid concentration remained consistent with that of the RT-qPCR assay.

### 3.6 Repeatability and reproducibility of the SVA RAA-LFD assay

**Figure 7:**
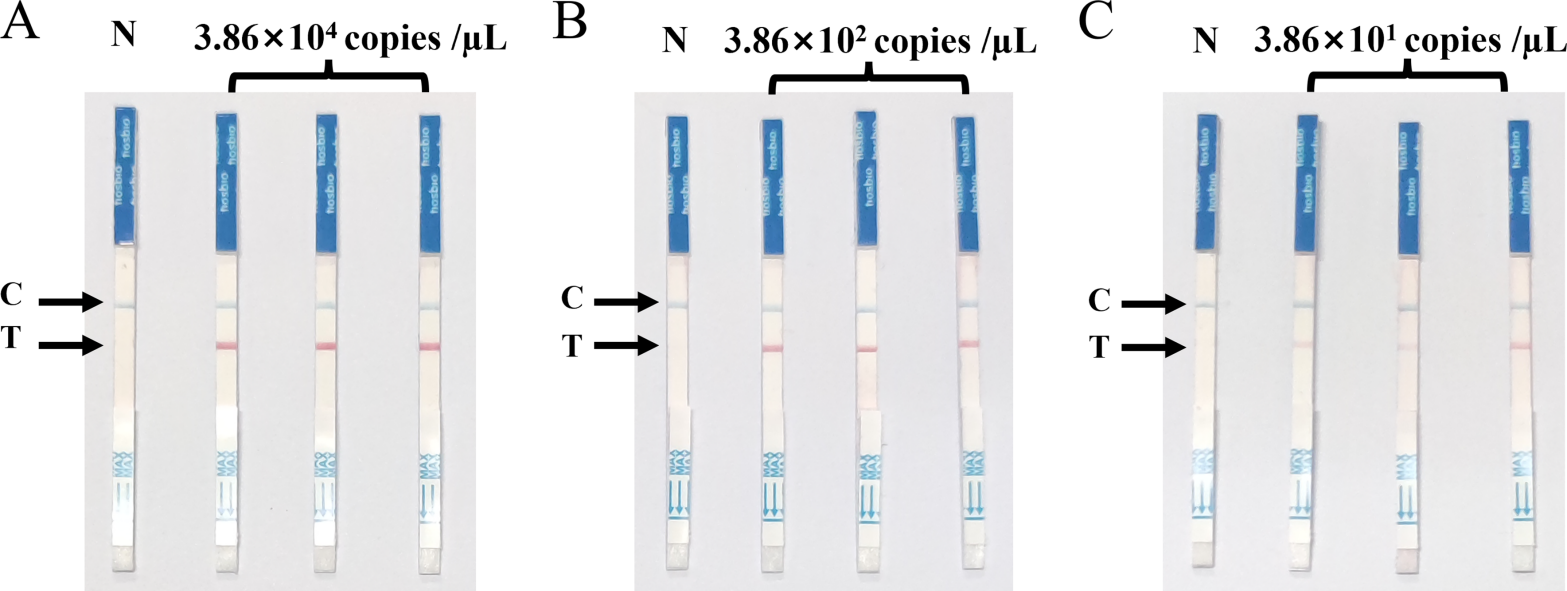
Repeatability of the SVA RAA-LFD assay. **(A)** Strong positive duplicate test of the SVA RAA-LFD assay for plasmid concentration of 3.86×10^4^ copies/µL. **(B)** Moderate positive duplicate test of the SVA RAA-LFD assay for plasmid concentration of 3.86×10^2^ copies/µL. **(C)** Weak positive duplicate test of the SVA RAA-LFD assay for plasmid concentration of 3.86×10^1^ copies/µL.

The SVA RAA-LFD assay for three different concentrations of the pMD18-SVA-3D plasmid showed all positive reaction signals at the T-line, with good reproducibility across three replicates. There was a slight difference in the intensity of the detection line positive response signal for the lowest detection concentration repeatability experiments, but all three replicates resulted in positive results (**Figure 7**).

### 3.7 Diagnostic performance of the SVA RAA-LFD assay evaluated with clinical swine samples

**Figure 8:**
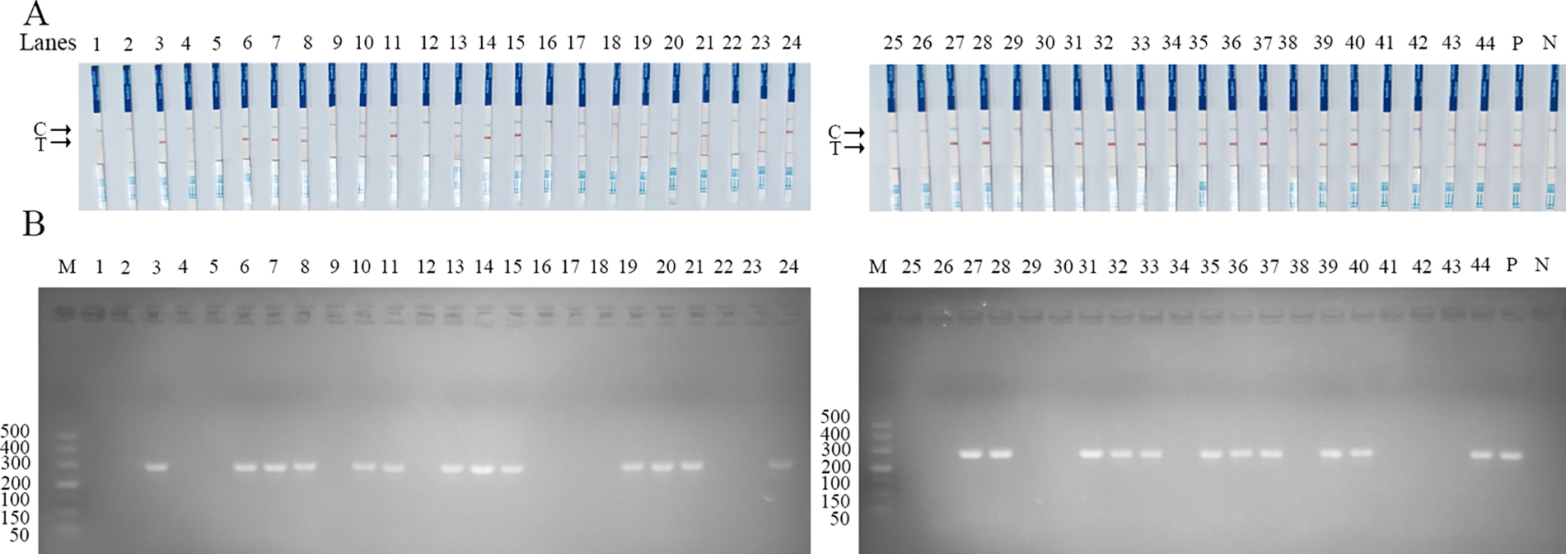
Performance of SVA RAA-LFD on clinical swine samples. **(A)** The result of clinical swine samples detection by the SVA RAA-LFD assay. **(B)** The result of clinical swine samples detection by conventional PCR method. Lanes1–44 Different clinical swine samples were preserved in our laboratory. N, Negative control; P, Positive control; M, 500 bp DNA marker.

To further assess the clinical performance of the SVA RAA-LFD assay, forty-four clinical samples were tested using the assay. Parallel experiments were conducted using PCR and RT-qPCR methods. The results of the PCR and RAA-LFD methods are presented in **Figure 8**, while the comparative outcomes of these three methods are displayed in **Table 2**. Specifically, the RAA-LFD method successfully detected 27 positive samples, the RT-qPCR method identified 27 samples, and the PCR method identified 24 samples. Notably, the RAA-LFD and RT-qPCR methods exhibited consistent detection rates, demonstrating the clinical feasibility of the SVA RAA-LFD assay in this study.

**Table 2:** Clinical application of the SVA RAA-LFD assay.

Detection method	Positive samples	Negative samples	Positive rate (%)
RAA-LFD	27	17	61
PCR	24	20	54
RT-qPCR	27	17	61

## 4 Discussion

SVA is a recently emerged pathogen of pigs that is both pathogenic and infectious, and it is clinically indistinguishable from other pathogens causing vesicular lesions in pigs, including FMDV. Furthermore, contemporary SVA isolates exhibit significant genetic divergence and evolution compared to historical strains, demonstrating a trend toward greater pathogenicity (Chen et al., 2019; Fernandes et al., 2018; Jiang et al., 2021; Joshi et al., 2016, 2020; Li et al., 2024; Wang et al., 2019; Zhang et al., 2020). Early diagnosis of SVA is critical not only for providing valuable epidemiological information and for the rapid initiation of prevention and control strategies but also for facilitating the accurate diagnosis, prevention, and control of other porcine vesicular epidemics, particularly FMD. Therefore, developing a highly specific and sensitive rapid diagnostic method for SVA is essential to provide effective technical support for these needs.

RAA is a novel molecular biology assay that allows for the amplification of large amounts of target nucleic acids in a short period, which has been widely used in the detection of a variety of pathogens that infect livestock or humans (Chen et al., 2022; Fan et al., 2020; Liu et al., 2023; Tian et al., 2023; Zhao et al., 2024a). In this study, we first established a rapid visualization assay specific to SVA by combining RAA with LFD. Initially, to ensure optimal sensitivity and specificity during the development of the SVA RAA-LFD assay, we identified the conserved region of the SVA *3D* gene as the target sequence by aligning the nucleotides of multiple sequences obtained from GenBank. We successfully screened for the optimal primer pair and designed the nfo-probe with the highest match.

Furthermore, building upon the preliminary SVA RAA-LFD assay, we fine-tuned the reaction time and temperature parameters. This adjustment allows the assay to be completed in just 17 minutes at 35 °C, comprising 15 minutes for the RAA basic reaction and 2 minutes for LFD processing. These modifications enhance the accuracy and efficiency of the developed assay. Subsequently, we further evaluated the specificity and sensitivity of the optimized assay. The final detection data showed that the lowest detection limits of the established assay for pMD18-SVA-3D plasmid, DNA amplification product, and viral were 3.86×10^1^cpoies/µL, 8.76×10^-7^ ng/µL, and 1×10^0.25^ TCID_50_/mL, respectively. The SVA RAA-LFD assay we developed is 100 times more sensitive to plasmids than the SVA *3D*-based PCR method studied by Qian et al. (Qian et al., 2016) and nearly consistent with the *VP1*-based RT-qPCR studied by Bracht et al (Bracht et al., 2016).

To assess clinical applicability, we tested forty-four clinical samples using the RAA-LFD assay, which showed a positive detection rate consistent with RT-qPCR. In comparison with the other two detection methodologies, the PCR assay detected three fewer positive samples. This discrepancy may arise from the low viral load present in the clinical samples, which could be beneath the PCR assay’s limit of detection, or it may be attributable to the primers’ insufficient specificity for the identification of SVA within the clinical samples. The RAA-LFD assay established in this study produced T-line specific positive reaction signals only for SVA, with no cross-reactivity to other common swine pathogens, including FMD, and demonstrated good reproducibility. In summary, the developed SVA RAA-LFD assay exhibits excellent sensitivity, specificity, and clinical applicability, making it a promising method for detecting SVA.

It is well known that the primary advantage of RAA is that it does not require an expensive thermal cycler to amplify the target gene. Compared to standard laboratory molecular biology detection techniques such as PCR and qPCR, RAA is more convenient and time-efficient, requiring only a water bath or a portable suitcase lab for operation, thereby enabling on-site diagnosis of the target gene (El Wahed et al., 2021). In recent years, other isothermal amplification techniques, such as LAMP, have been used for SVA detection (Armson et al., 2019; Zeng et al., 2018). However, compared to LAMP, RAA offers two distinct advantages. First, while LAMP necessitates a reaction temperature range of 60~65°C, RAA operates efficiently at a lower temperature, approximately 37°C. In our study, target gene amplification occurred within a temperature range of 34~40°C, with 35°C being the most favorable; notably, the reaction can even proceed at body temperature. Second, LAMP requires 4~6 primers to support the amplification reaction. In contrast, the RAA reaction can be accomplished with just two conventional primers or by adding a specific probe.

The combined application of RAA and LFD integrates the high sensitivity of polymerase chain reaction with immunochromatographic techniques, enabling rapid visualization of detection results (Homklinkaew et al., 2023; Li et al., 2022; Yu et al., 2019). In brief, this method involves the combination of target genes labeled with specific probes with antibodies conjugated to colloidal gold encapsulated on the test strip, ultimately translating the detection results into visible color signals discernible to the naked eye. The simplicity of operation and lack of requirement for expensive instrumentation make this method suitable for application in remote areas and field settings.

Regrettably, the method established in this study was not validated with clinical samples from multiple countries. However, previous studies have shown that the RAA reaction can accommodate mismatches of 5-9 nucleotide bases (Abd El. Wahed et al., 2013). Upon aligning multiple SVA sequences from various countries, including China, the USA, and Brazil, we found that the individual base differences in the selected amplification sequences were less than 5 nucleotides, indicating the potential applicability of the assay used in this study. Testing a large variety of clinical swine samples would provide more robust evidence for the feasibility of the developed method. Therefore, we intend to seek out multiple clinical samples for further validation in subsequent investigations.

In summary, this study has established a convenient and sensitive RAA-LFD assay based on the conserved region of the SVA*3D* gene. The assay provides visually detectable results in under 17 minutes using an instrument-free technique. This makes it suitable for point-of-care diagnosis for SVA in the field or remote areas lacking instrumentation.

## Data Availability

The original contributions presented in the study are included in the article/**Supplementary Material**. Further inquiries can be directed to the corresponding authors.

## References

[B1] Abd El. Wahed A. El-Deeb A. El-Tholoth M. Abd El Kader H. Ahmed A. Hassan S.. (2013). A portable reverse transcription recombinase polymerase amplification assay for rapid detection of foot-and-mouth disease virus. PloS One 8, e71642. doi: 10.1371/journal.pone.0071642 23977101 PMC3748043

[B2] Adams M. J. Lefkowitz E. J. King A. M. Q. Bamford D. H. Breitbart M. Davison A. J.. (2015). Ratification vote on taxonomic proposals to the International Committee on Taxonomy of Viruses. Arch. Virol. 160, 1837–1850. doi: 10.1007/s00705-015-2425-z 25913692

[B3] Adams M. J. Lefkowitz E. J. King A. M. Harrach B. Harrison R. L. Knowles N. J.. (2016). Ratification vote on taxonomic proposals to the International Committee on Taxonomy of Viruses. Arch. Virol. 161, 2921–2949. doi: 10.1007/s00705-016-2977-6 27424026 PMC7086986

[B4] Armson B. Walsh C. Morant N. Fowler V. L. Knowles N. J. Clark D. (2019). The development of two field-ready reverse transcription loop-mediated isothermal amplification assays for the rapid detection of Seneca Valley virus 1. Transbound Emerg. Dis. 66, 497–504. doi: 10.1111/tbed.13051 30372584 PMC6434928

[B5] Arzt J. Bertram M. R. Vu L. T. Pauszek S. J. Hartwig E. J. Smoliga G. R.. (2019). First detection and genome sequence of senecavirus A in Vietnam. Microbiol. Resour. Announc. 8, e01247–e01218. doi: 10.1128/MRA.01247-18 30687818 PMC6346150

[B6] Bienes K. M. Mao L. Selekon B. Gonofio E. Nakoune E. Wong G.. (2022). Rapid detection of the varicella-zoster virus using a recombinase-aided amplification-lateral flow system. Diagnostics (Basel) 12, 2957. doi: 10.3390/diagnostics12122957 36552964 PMC9777233

[B7] Bracht A. J. O’Hearn E. S. Fabian A. W. Barrette R. W. Sayed A. (2016). Real-time reverse transcription PCR assay for detection of senecavirus A in swine vesicular diagnostic specimens. PloS One 11, e0146211. doi: 10.1371/journal.pone.0146211 26757142 PMC4710529

[B8] Chen L. Zhang J. Wang M. Pan S. Mou C. Chen Z. (2019). Pathogenicity of two Chinese Seneca Valley virus (SVV) strains in pigs. Microbial pathogenesis. 136, 103695. doi: 10.1016/j.micpath.2019.103695 31449854

[B9] Chen Y. Zong N. Ye F. Mei Y. Qu J. Jiang X. (2022). Dual-CRISPR/cas12a-assisted RT-RAA for ultrasensitive SARS-coV-2 detection on automated centrifugal microfluidics. Analytical Chem. 94, 9603–9609. doi: 10.1021/acs.analchem.2c00638 35775831

[B10] Chow W. H. McCloskey C. Tong Y. Hu L. You Q. Kelly C. P.. (2008). Application of isothermal helicase-dependent amplification with a disposable detection device in a simple sensitive stool test for toxigenic Clostridium difficile. J. Mol. Diagn. 10, 452–458. doi: 10.2353/jmoldx.2008.080008 18669881 PMC2518740

[B11] Dvorak C. M. Akkutay-Yoldar Z. Stone S. R. Tousignant S. J. Vannucci F. A. Murtaugh M. P. (2017). An indirect enzyme-linked immunosorbent assay for the identification of antibodies to Senecavirus A in swine. BMC veterinary Res. 13, 50. doi: 10.1186/s12917-017-0967-x PMC5312445 28202026

[B12] El Wahed A. A. Patel P. Maier M. Pietsch C. Rüster D. Böhlken-Fascher S.. (2021). Suitcase lab for rapid detection of SARS-coV-2 based on recombinase polymerase amplification assay. Anal. Chem. 93, 2627–2634. doi: 10.1021/acs.analchem.0c04779 33471510 PMC7839158

[B13] Fan X. Li L. Zhao Y. Liu Y. Liu C. Wang Q.. (2020). Clinical validation of two recombinase-based isothermal amplification assays (RPA/RAA) for the rapid detection of African swine fever virus. Front. Microbiol. 11, 1696. doi: 10.3389/fmicb.2020.01696 32793160 PMC7385304

[B14] Feng J. Chen J. Du B. Cui X. Xia Y. Xue G.. (2024). Development of a recombinase-aided amplification assay for the rapid detection of candida auris. Anal. Chem. 96, 9424–9429. doi: 10.1021/acs.analchem.4c00450 38825761

[B15] Fernandes M. H. V. Maggioli M. F. Joshi L. R. Clement T. Faccin T. C. Rauh R.. (2018). Pathogenicity and cross-reactive immune responses of a historical and a contemporary Senecavirus A strains in pigs. Virology. 522, 147–157. doi: 10.1016/j.virol.2018.06.003 30029014

[B16] Hai C. (2010). Recombinase-aid amplification:a novel technology of in *vitro* rapid nucleic acid amplification. Scientia Sinica (Vitae). 40, 983–988.

[B17] Hales L. M. Knowles N. J. Reddy P. S. Xu L. Hay C. Hallenbeck P. L. (2008). Complete genome sequence analysis of Seneca Valley virus-001, a novel oncolytic picornavirus. J. Gen. Virol. 89, 1265–1275. doi: 10.1099/vir.0.83570-0 18420805

[B18] Hause B. M. Myers O. Duff J. Hesse R. A. (2016). Senecavirus A in pigs, United States 2015. Emerg. Infect. Dis. 22, 1323–1325. doi: 10.3201/eid2207.151591 27314580 PMC4918151

[B19] He Y. Chen W. Fan J. Fan S. Ding H. Chen J.. (2021). Recombinase-aided amplification coupled with lateral flow dipstick for efficient and accurate detection of porcine parvovirus. Life (Basel) 11, 762. doi: 10.3390/life11080762 34440506 PMC8401844

[B20] Homklinkaew P. Phatthanakunanan S. Jala S. Boonsoongnern A. Lertwatcharasarakul P. (2023). Development of a recombinase-aided amplification method combined with lateral flow dipstick assay to detect Porcine circovirus type 2. Vet. World 16, 2313–2320. doi: 10.14202/vetworld. 38152256 PMC10750741

[B21] Hou L. Li D. Zhang N. Zhao J. Zhao Y. Sun X. (2022). Development of an isothermal recombinase-aided amplification assay for the rapid and visualized detection of Klebsiella pneumoniae. J. Sci. Food Agric. 102, 3879–3886. doi: 10.1002/jsfa.v102.9 34936095

[B22] Jaroenram W. Kiatpathomchai W. Flegel T. W. (2009). Rapid and sensitive detection of white spot syndrome virus by loop-mediated isothermal amplification combined with a lateral flow dipstick. Mol. Cell Probes 23, 65–70. doi: 10.1016/j.mcp.2008.12.003 19124071

[B23] Jaroenram W. Owens L. (2014). Recombinase polymerase amplification combined with a lateral flow dipstick for discriminating between infectious Penaeus stylirostris densovirus and virus-related sequences in shrimp genome. J. Virol. Methods 208, 144–151. doi: 10.1016/j.jviromet.2014.08.006 25152528

[B24] Jiang J. Zha Y. Liu J. Xing C. Mi S. Yu J.. (2021). Isolation and evolutionary analysis of Senecavirus A isolates from Guangdong province, China. Infect. Genet. Evol. 91, 104819. doi: 10.1016/j.meegid.2021.104819 33771724

[B25] Joshi L. R. Fernandes M. H. V. Clement T. Lawson S. Pillatzki A. Resende T. P.. (2016). Pathogenesis of Senecavirus A infection in finishing pigs. J. Gen. Virol. 97, 3267–3279. doi: 10.1099/jgv.0.000631 27902357

[B26] Joshi L. R. Mohr K. A. Gava D. Kutish G. Buysse A. S. Vannucci F. A.. (2020). Genetic diversity and evolution of the emerging picornavirus Senecavirus A. J. Gen. Virol. 101, 175–187. doi: 10.1099/jgv.0.001360 31859611

[B27] K. S. S. C. G. C. S. G. S. R. F. (2012). Seneca valley virus and vesicular lesions in a pig with idiopathic vesicular disease. J. Vet. Sci. 03, 123. doi: 10.4172/2157-7579.1000123

[B28] King A. M. Q. Adams M. J. Carstens E. B. Lefkowitz E. J. (2012). Virus taxonomy: ninth report of the international committee on taxonomy of viruses. Arch. Virol. 140, 1221–1234. doi: 10.1007/BF01309873

[B29] Knowles N. J. Hales L. M. Jones B. H. Landgraf J. G. Hallenbeck P. L. (2006). Epidemiology of seneca valley virus: identification and characterization of isolates from pigs in the United States.

[B30] Kolm C. Martzy R. Führer M. Mach R. L. Krska R. Baumgartner S.. (2019). Detection of a microbial source tracking marker by isothermal helicase-dependent amplification and a nucleic acid lateral-flow strip test. Sci. Rep. 9, 393. doi: 10.1038/s41598-018-36749-7 30674936 PMC6344534

[B31] Leme R. A. Miyabe F. M. Dall Agnol A. M. Alfieri A. F. Alfieri A. A. (2019). A new wave of Seneca Valley virus outbreaks in Brazil. Transbound Emerg. Dis. 66, 1101–1104. doi: 10.1111/tbed.2019.66.issue-3 30771273

[B32] Leme R. A. Zotti E. Alcântara B. K. Oliveira M. V. Freitas L. A. Alfieri A. F.. (2015). Senecavirus A: an emerging vesicular infection in Brazilian pig herds. Transbound Emerg. Dis. 62, 603–611. doi: 10.1111/tbed.2015.62.issue-6 26398783

[B33] Li J. S. Hao Y. Z. Hou M. L. Zhang X. Zhang X. G. Cao Y. X.. (2022). Development of a recombinase-aided amplification combined with lateral flow dipstick assay for the rapid detection of the African swine fever virus. BioMed. Environ. Sci. 35, 133–140. doi: 10.3967/bes2022.018 35197178

[B34] Li J. Liang W. Xu S. Shi J. Zhou X. Liu B.. (2019). Rapid and sensitive detection of Senecavirus A by reverse transcription loop-mediated isothermal amplification combined with a lateral flow dipstick method. PloS One 14, e0216245. doi: 10.1371/journal.pone.0216245 31048910 PMC6497277

[B35] Li Y. Liu T. Zhang Y. Duan X. Liu F. (2024). RNA recombination: non-negligible factor for preventing emergence or reemergence of Senecavirus A. Front. Vet. Sci. 11, 1357179. doi: 10.3389/fvets.2024.1357179 38328259 PMC10847583

[B36] Li Y. Shang J. Wang Y. Luo J. Jiang W. Yin X.. (2023). Establishment of two assays based on reverse transcription recombinase-aided amplification technology for rapid detection of H5 subtype avian influenza virus. Microbiol. Spectr. 11, e0218623. doi: 10.1128/spectrum.02186-23 37811963 PMC10715165

[B37] Lin J. Y. Chen T. C. Weng K. F. Chang S. C. Chen L. L. Shih S. R. (2009). Viral and host proteins involved in picornavirus life cycle. J. Biomed. Sci. 16, 103. doi: 10.1186/1423-0127-16-103 19925687 PMC2785775

[B38] Liu W. Zhang G. Xu D. Ye J. Lu Y. (2023). A novel RAA combined test strip method based on dual gene targets for pathogenic vibrio vulnificus in aquatic products. Foods 12, 3605. doi: 10.3390/foods12193605 37835259 PMC10572794

[B39] Mu S. Abdullah S. W. Zhang Y. Han S. Guo H. Li M.. (2020). Development of a novel SYBR green I-based quantitative RT-PCR assay for Senecavirus A detection in clinical samples of pigs. Mol. Cell Probes. 53, 101643. doi: 10.1016/j.mcp.2020.101643 32768439

[B40] Pasma T. Davidson S. Shaw S. L. (2008). Idiopathic vesicular disease in swine in Manitoba. Can. Vet. J. 49, 84–85. 18320985 PMC2147704

[B41] Peng Y. Hu R. Xue S. He Y. Tian L. Pang Z.. (2024). Rapid and highly sensitive colorimetric LAMP assay and integrated device for visual detection of monkeypox virus. Anal. Chim. Acta 1311, 342720. doi: 10.1016/j.aca.2024.342720 38816155

[B42] Pinheiro-de-Oliveira T. F. Fonseca-Júnior A. A. Camargos M. F. Laguardia-Nascimento M. Giannattasio-Ferraz S. Cottorello A. C. P.. (2019). Reverse transcriptase droplet digital PCR to identify the emerging vesicular virus Senecavirus A in biological samples. Transbound Emerg. Dis. 66, 1360–1369. doi: 10.1111/tbed.13168 30864242

[B43] Qian S. Fan W. Qian P. Chen H. Li X. (2016). Isolation and full-genome sequencing of Seneca Valley virus in piglets from China 2016. Virol. J. 13, 173. doi: 10.1186/s12985-016-0631-2 27756396 PMC5069920

[B44] Saeng-chuto K. Rodtian P. Temeeyasen G. Wegner M. Nilubo D. (2017). The first detection of Senecavirus A in pigs in Thailand 2016. Transbound Emerg. Dis. 65, 285–288. doi: 10.1111/tbed.12654 28474854

[B45] Sun D. Vannucci F. Knutson T. P. Corzo C. Marthaler D. G. (2017). Emergence and whole-genome sequence of Senecavirus A in Colombia. Transbound Emerg. Dis. 64, 1346–1349. doi: 10.1111/tbed.2017.64.issue-5 28714178

[B46] Tian Y. Fan Z. Xu L. Cao Y. Chen S. Pan Z.. (2023). CRISPR/Cas13a-assisted rapid and portable HBV DNA detection for low-level viremia patients. Emerg. Microbes Infect. 12, e2177088. doi: 10.1080/22221751.2023.2177088 36735916 PMC9946317

[B47] Vannucci F. A. Linhares D. C. Barcellos D. E. Lam H. C. Collins J. Marthaler D. (2015). Identification and complete genome of seneca valley virus in vesicular fluid and sera of pigs affected with idiopathic vesicular disease, Brazil. Transbound Emerg. Dis. 62, 589–593. doi: 10.1111/tbed.2015.62.issue-6 26347296

[B48] Venkataraman S. Reddy S. P. Loo J. Idamakanti N. Hallenbeck P. L. Reddy V. S. (2008). Structure of Seneca Valley Virus-001: an oncolytic picornavirus representing a new genus. Structure 16, 1555–1561. doi: 10.1016/j.str.2008.07.013 18940610 PMC2572565

[B49] Vieira M. V. Yasumitsu C. Y. Dall Agnol A. M. Leme R. A. Alfieri A. F. Alfieri A. A. (2022). The third wave of Seneca Valley virus outbreaks in pig herds in southern Brazil. Braz. J. Microbiol. 53, 1701–1706. doi: 10.1007/s42770-022-00767-5 35554870 PMC9433486

[B50] Wang M. Chen L. Pan S. Mou C. Shi K. Chen Z. (2019). Molecular evolution and characterization of novel Seneca Valley virus (SVV) strains in South China. Infect. Genet. Evol. 69, 1–7. doi: 10.1016/j.meegid.2019.01.004 30639519

[B51] Wang Q. Heo W. Choi S. Jang W. Lim C. S. Jung H. I. (2024). Hand-held all-in-one (HAO) self-test kit for rapid and on-site detection of SARS-CoV-2 with colorimetric LAMP. Lab. Chip 24, 3265–3275. doi: 10.1039/D4LC00199K 38847067

[B52] Wu X. Liu Y. Gao L. Yan Z. Zhao Q. Chen F.. (2022). Development and application of a reverse-transcription recombinase-aided amplification assay for porcine epidemic diarrhea virus. Viruses 14, 591. doi: 10.3390/v14030591 35336998 PMC8948910

[B53] Wu Q. Zhao X. Bai Y. Sun B. Xie Q. Ma J. (2017). The first identification and complete genome of senecavirus A affecting pig with idiopathic vesicular disease in China. Transbound Emerg. Dis. 64, 1633–1640. doi: 10.1111/tbed.2017.64.issue-5 27539949

[B54] Xu W. Hole K. Goolia M. Pickering B. Nfon C. (2017). Genome wide analysis of the evolution of Senecavirus A from swine clinical material and assembly yard environmental samples. PloS One 12, e0176964. doi: 10.1371/journal.pone.0176964 28475630 PMC5419577

[B55] Yang M. van Bruggen R. Xu W. (2012). Generation and diagnostic application of monoclonal antibodies against Seneca Valley virus. J. Vet. Diagn. Invest. 24, 42–50. doi: 10.1177/1040638711426323 22362934

[B56] Yu J. Shen D. Dai T. Lu X. Xu H. Dou D. (2019). Rapid and equipment-free detection of Phytophthora capsici using lateral flow strip-based recombinase polymerase amplification assay. Lett. Appl. Microbiol. 69, 64–70. doi: 10.1111/lam.13166 31021437

[B57] Zeng F. Cong F. Liu X. Lian Y. Wu M. Xiao L.. (2018). Development of a real time loop-mediated isothermal amplification method for detection of Senecavirus A. J. Virol. Methods 261, 98–103. doi: 10.1016/j.jviromet.2018.08.005 30096349

[B58] Zhang H. Chen P. Hao G. Liu W. Chen H. Qian P.. (2020). Comparison of the pathogenicity of two different branches of senecavirus a strain in China. Pathogens 9, 39. doi: 10.3390/pathogens9010039 31906571 PMC7168630

[B59] Zhang Z. Zhang Y. Lin X. Chen Z. Wu S. (2019). Development of a novel reverse transcription droplet digital PCR assay for the sensitive detection of Senecavirus A. Transbound Emerg. Dis. 66, 517–525. doi: 10.1111/tbed.2019.66.issue-1 30375741

[B60] Zhao Y. Yang M. Zhou C. Guo B. Wang K. Song C.. (2024a). Establishment of a simple, sensitive, and specific ASFV detection method based on Pyrococcus furiosus argonaute. Biosens. Bioelectron. 254, 116230. doi: 10.1016/j.bios.2024.116230 38520983

[B61] Zhao Y. Zhou C. Guo B. Yang X. Wang H. (2024b). Pyrococcus furiosus Argonaute-mediated porcine epidemic diarrhea virus detection. Appl. Microbiol. Biotechnol. 108, 137. doi: 10.1007/s00253-023-12919-0 38229331 PMC10789834

[B62] Zhou C. Zhao Y. Guo B. Yang M. Xu Q. Lei C.. (2024). Establishment of a simple, sensitive, and specific salmonella detection method based on recombinase-aided amplification combined with dsDNA-specific nucleases. Foods 13, 1380. doi: 10.3390/foods13091380 38731750 PMC11083397

