# Corrigendum: A rapid and visual detection assay for Senecavirus A based on recombinase-aided amplification and lateral flow dipstick

**DOI:** 10.3389/fcimb.2024.1526755

**Published:** 2024-12-04

**Authors:** Yiwan Song, Yiqi Fang, Shuaiqi Zhu, Weijun Wang, Lianxiang Wang, Wenxian Chen, Yintao He, Lin Yi, Hongxing Ding, Mingqiu Zhao, Shuangqi Fan, Zhaoyao Li, Jinding Chen

**Affiliations:** ^1^ College of Veterinary Medicine, South China Agricultural University, Guangzhou, China; ^2^ Key Laboratory of Zoonotic Disease Prevention and Control of Guangdong, South China Agricultural University, Guangzhou, China; ^3^ Wen’s Group Academy, Wen’s Foodstuffs Group Co., Ltd., Guangzhou, Guangdong, China

**Keywords:** Senecavirus A, recombinase-aided amplification, lateral flow dipstick, sensitivity, specificity, visual detection

In the published article, there was an error regarding the affiliation for Zhaoyao Li. As well as having affiliation(s) 3, they should also have “^1^College Veterinary Medicine, South China Agricultural University, Guangzhou, China”.

The authors apologize for this error and state that this does not change the scientific conclusions of the article in any way. The original article has been updated.

