# Supplementary material for: A rapid and visual detection assay for Senecavirus A based on recombinase-aided amplification and lateral flow dipstick
Source: Front Cell Infect Microbiol. 2024 Oct 23;14:1474676. doi: 10.3389/fcimb.2024.1474676 (PMC11538013; doi:10.3389/fcimb.2024.1474676)
Supplement: Supplementary file 1 [file SupplementaryFile1.docx]

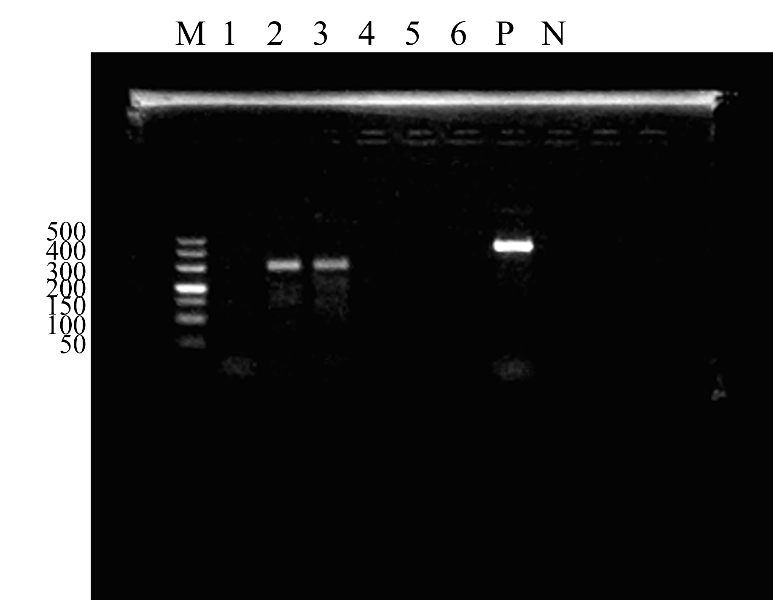
Supplementary Material: Original gel images


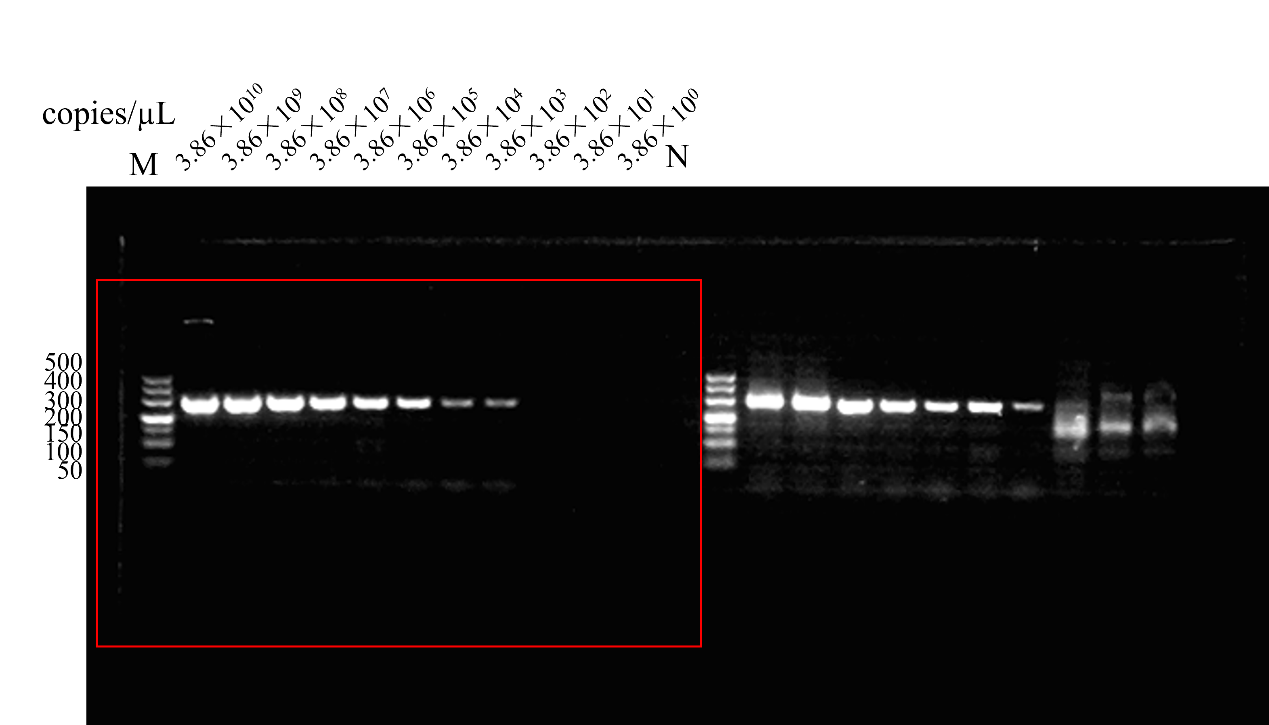
FIGURE S1 The original gel images of FIGURE 2.

FIGURE S2 The original gel images of FIGURE 6A. All cropped gel image parts in the manuscript are highlighted with red frames on the original gel images.


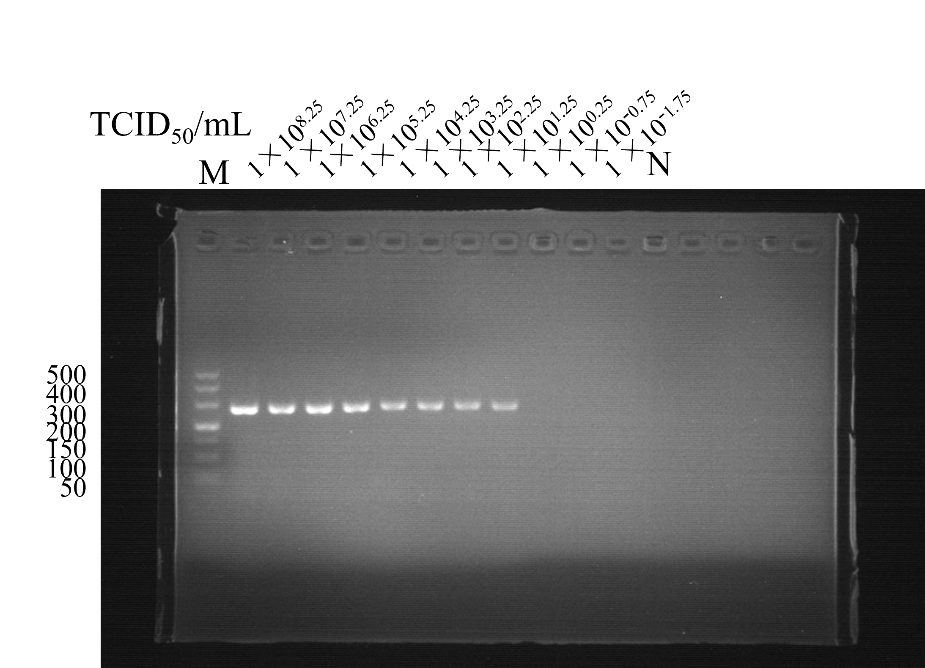
FIGURE S3 The original gel images of FIGURE 6B.
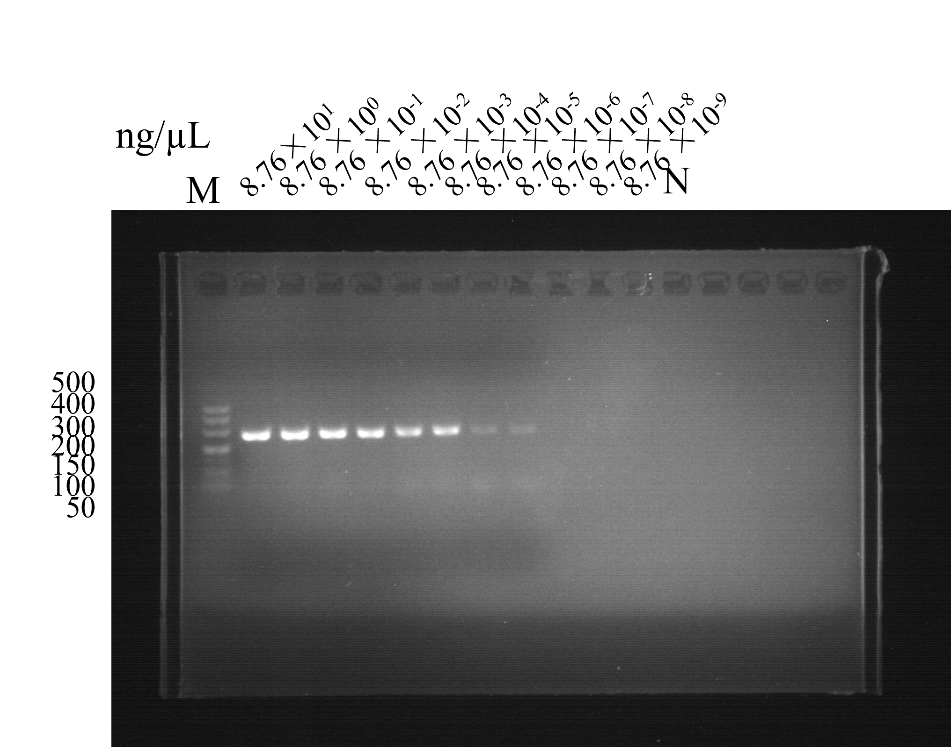


FIGURE S4 The original gel images of FIGURE 6C.


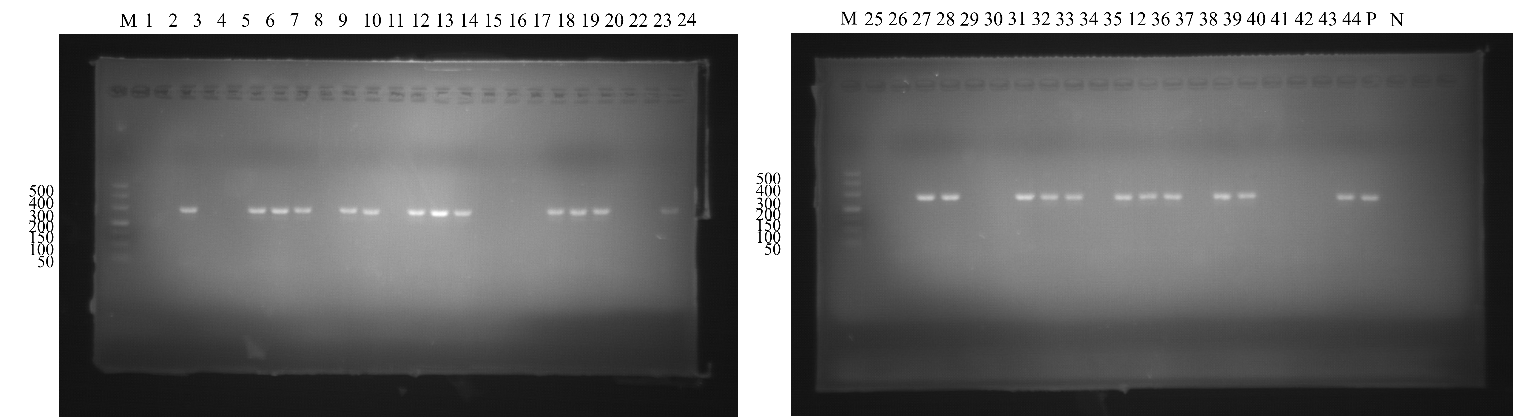
FIGURE S5 The original gel images of FIGURE 8.
